# From Particles to Chemicals: Redefining the Impact of Agricultural Plastics on Water Quality

**DOI:** 10.1021/acs.est.6c05931

**Published:** 2026-06-08

**Authors:** Wei Chen, Thilo Hofmann

**Affiliations:** † College of Environmental Science and Engineering, Ministry of Education Key Laboratory of Pollution Processes and Environmental Criteria, Tianjin Key Laboratory of Environmental Remediation and Pollution Control, 12538 Nankai University, Tianjin 300350, China; ‡ Department of Environmental Geosciences (EDGE), Centre for Microbiology and Environmental Systems Science, 27258 University of Vienna, 1090 Vienna, Austria

**Keywords:** agricultural plastics, environmental footprint, water quality, degradation products

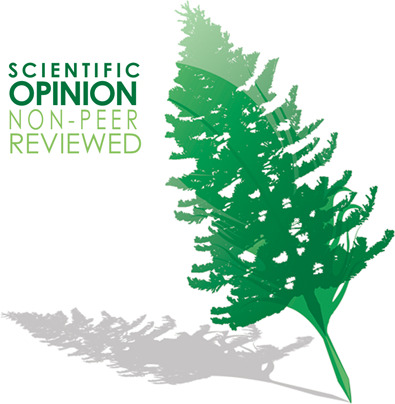

## Graphical abstract

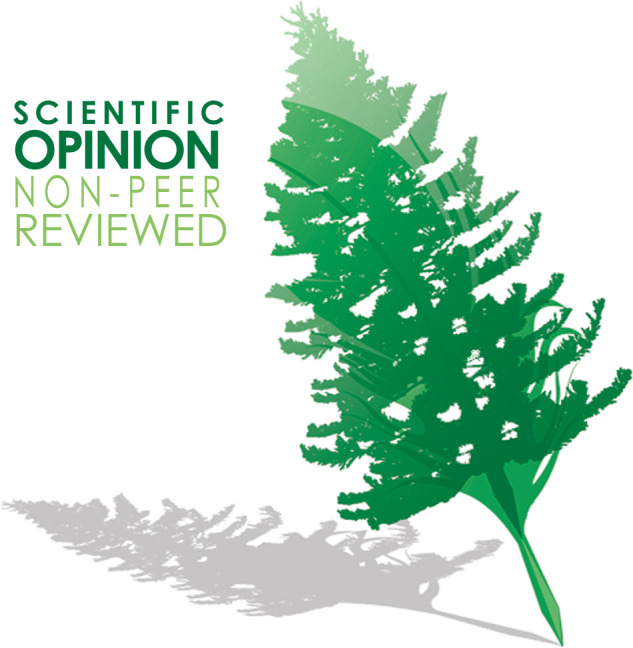

Agricultural plastics have become indispensable in modern crop production by improving water use efficiency, stabilizing yields, and reducing agrochemical inputs.^1^ Global agricultural plastic use is estimated to exceed 12 million t annually, with mulch films representing a major application worldwide.^2^ As a result, plastic materials such as polyethylene (PE), polypropylene (PP), polyvinyl chloride (PVC), and ethylene-vinyl acetate (EVA) are now widely embedded in agricultural systems. Given their agronomic benefits, agricultural plastics as a whole are unlikely to be replaced at scale.^3^ Thus, their widespread and persistent use makes understanding their environmental footprint a central priority, particularly in the context of water quality, as agricultural soils act as key interfaces that control contaminant fluxes to groundwater, surface waters, and ultimately drinking water systems.^3^ To date, however, assessments have focused predominantly on the formation and accumulation of micro- and nanoplastic particles in soils, overlooking potentially more relevant pathways of environmental exposure.

We argue that the dominant impact of agricultural plastics on water quality may not arise from the emissions of micro- and nanoplastics themselves, but from their role as long-term reservoirs that release embedded chemicals and degradation products. All agricultural plastics contain diverse chemical additives, including plasticizers, UV stabilizers, antioxidants, pigments, and other processing agents. For example, additive fractions can reach 10% by weight in polyolefin-based films, whereas the plasticizer content in PVC components often exceeds 20–35% by weight.^4,5^ Although the weathering and fragmentation of agricultural plastics generate substantial amounts of plastic residues, these particles tend to accumulate predominantly within the unsaturated zone.^6,7^ In contrast, a more prominent risk is the release of associated chemicals into soil systems, which act as reactive interfaces controlling the partitioning and transformation of these chemicals, and their transport to groundwater and surface waters. Safeguarding aquatic ecosystems therefore necessitates moving beyond particle-centric paradigms to explicitly address the chemical fluxes originating from agricultural plastics and entering water resources.

## Moving from Particles to Chemicals

Current environmental assessments are largely centered on the formation, persistence, and transport of micro- and nanoplastic particles in soils. Although fragmentation and accumulation are well established and represent a global concern, the role of these particles as mobile pollutants or effective contaminant carriers in soil–water systems appears to be limited. Physical filtration, straining, and interception strongly restrict particle transport, leading to effective retention of plastics within the upper soil horizon over short distances. While vertical transport to groundwater has been reported under specific conditions, such occurrences remain limited relative to the flux of dissolved chemicals.^6^ In addition, the widely cited “Trojan horse” mechanism may be less relevant in terrestrial environments, as sorption of inorganic and organic contaminants is dominated by soil mineral interfaces and soil organic matter rather than plastic surfaces.^8^ Together, these observations suggest that particle-based pathways alone do not adequately capture environmental exposure, particularly with regard to the impact on water quality. Rather, the impact is mostly driven by mobile plastic-derived chemicals, including additives and their transformation products.

All agricultural plastics, conventional and biodegradable, contain complex mixtures of additives, including plasticizers, UV stabilizers, antioxidants, and processing agents.^9^ Many commonly used additives, including phthalates, benzotriazoles, and bisphenol-related compounds, exhibit bioactive properties, including endocrine disruption and chronic toxicity. Because these additives are not chemically bound to the polymer matrix, their release is thermodynamically driven and inevitable. For intact polymers, additive leaching is diffusion-limited, resulting in slow but continuous emissions over long time scales.^10^ In contrast, degradation processes, such as photo-oxidation, mechanical fragmentation, or biodegradation, progressively disrupt the polymer matrix and thereby accelerate the release of embedded chemicals. These processes are further intensified by environmental stressors, including ultraviolet radiation, temperature fluctuations, and repeated wet–dry or thaw–freeze cycles. Thus, agricultural plastics function as long-term chemical reservoirs, an inherent consequence of their material design. From an exposure perspective, this results in a fundamental asymmetry. While particle transport is largely constrained by physical retention in soils and the aquifer itself, chemical emissions occur as continuous or episodic mass fluxes that are not subject to the same filtration and straining processes and can therefore dominate the transport of plastic-derived contaminants to groundwater and surface waters.

Importantly, these emissions are not limited to intact plastic materials. In practice, complete removal of agricultural plastics from soil is rarely achieved, as mechanical retrieval after use typically leaves behind substantial amounts of fragmented residues embedded within the soil matrix. From a chemical perspective, these residuals represent long-term sources, continuously releasing dissolved and potentially hazardous chemicals into the soil–water system. As a result, these residual plastics extend the temporal and spatial extent of chemical exposure well beyond the initial period and location of plastics application.

A similar shift in perspective is required when considering biodegradable agricultural plastics. Although these materials are designed to degrade more rapidly, this accelerated breakdown can enhance the release of embedded additives, along with soluble oligomers and monomers, into the soil–water system.^11^ To maintain functional performance during use, biodegradable materials often contain complex additive mixtures, including stabilizers, plasticizers, processing aids, and other intentionally or non-intentionally added substances.^12,13^ Consequently, rather than eliminating chemical emissions, biodegradable mulch films may shift the emission regime, from slow, diffusion-controlled release to more rapid and temporally concentrated pulses of mobile plastic-derived chemicals, challenging the assumption that biodegradability inherently reduces environmental risk.

Once released into the soil–water system, these chemicals transition from material-bound constituents to mobile solutes, where their transport is governed by environmental partitioning, transformation, and hydrological processes rather than physical filtration constraints. These pathways directly connect agricultural soils to receiving water bodies and drinking water resources, particularly in regions with shallow groundwater tables or intensive irrigation practices, allowing the chemicals to migrate beyond the immediate source zone. These emissions are not constant but are strongly influenced by hydrological conditions. Episodic events such as intense rainfall or flooding can mobilize accumulated chemicals from agricultural soils, generating short-term pulses of increased concentrations in receiving water bodies. Such pulse events may lead to transient but significant contamination of surface waters and groundwater recharge zones, with potential implications for downstream water treatment and drinking water quality. Given that plastic-derived chemicals span a wide range of chemical properties, conventional treatment processes may fail to effectively remove some of these constituents, allowing their occurrence in drinking water. At the scale of global agricultural plastic use, these continuous and event-driven emissions constitute a diffuse and sustained source of chemical inputs to aquatic systems, with implications ranging from ecosystem impacts to drinking water contamination and associated human exposure (Figure 1).

**Figure 1:**
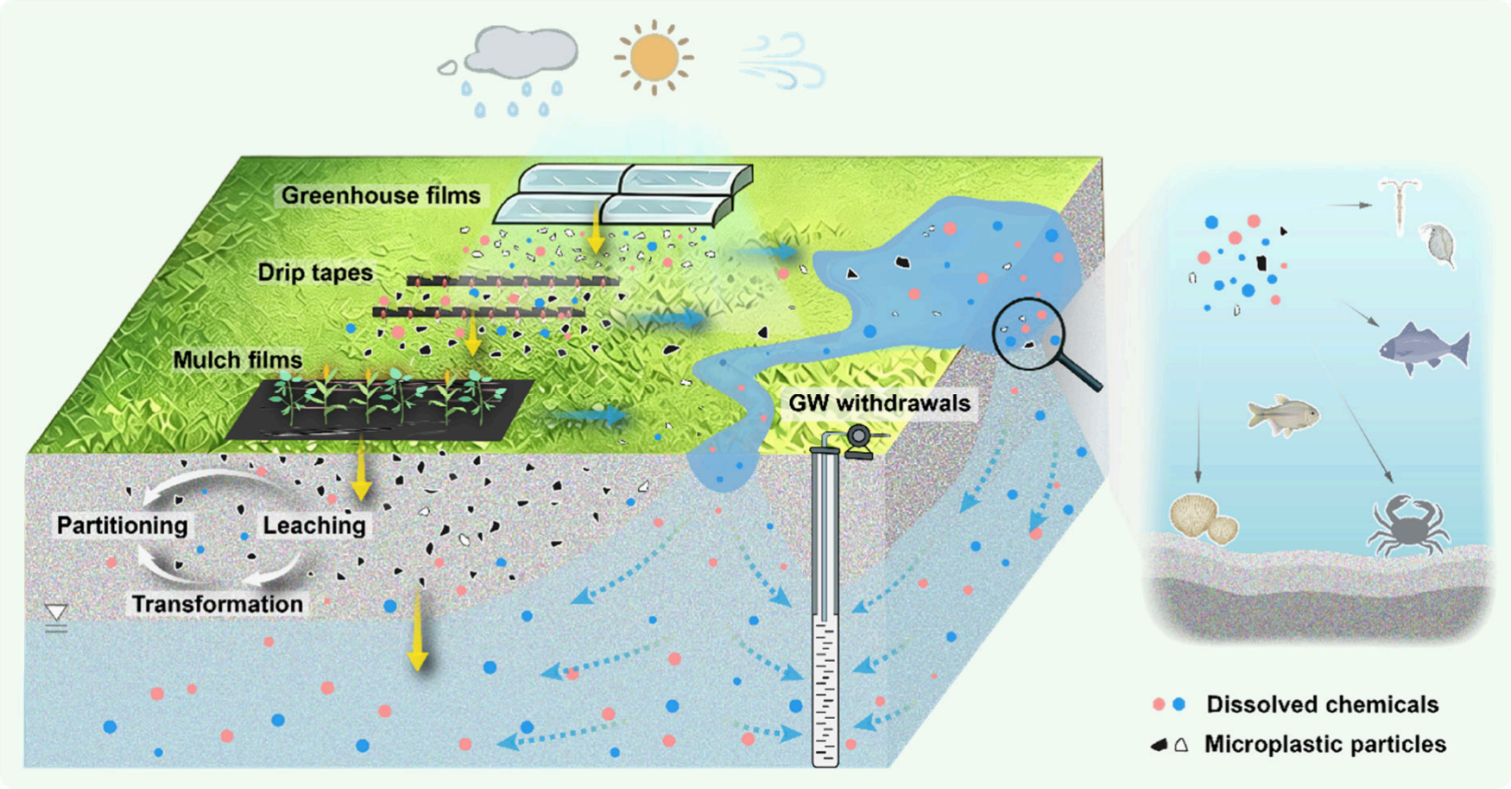
Agricultural plastics as sources of mobile plastic-derived chemicals in soil–water systems. The release of plastic-derived chemicals occurs via diffusion and degradation of plastic materials, followed by partitioning and transformation within the soil profile. These chemicals are subsequently transported to groundwater and surface waters, with hydrological events triggering pulsed emissions. While particle transport is largely constrained by retention in soils, the flux of mobile plastic-derived chemicals represents a key pathway for environmental exposure and water quality impacts.

## Blind Spots and Challenges

Despite its critical importance, the chemical dimension of agricultural plastics remains poorly constrained. The identities and concentrations of additives in commercial products are often not fully disclosed, and a substantial fraction of plastic-derived chemicals remains unknown. Furthermore, additive formulations are highly heterogeneous and application-specific, reflecting different agronomic, climatic, and material requirements.^14^ As a result, agricultural plastics introduce regionally distinct chemical signatures, complicating the development of generalized exposure and risk assessments across systems. In addition, plastics release evolving mixtures of parent additives and their transformation products, posing great analytical challenges. This problem is further exacerbated by complex environmental matrices, where trace-level detection of plastic-derived chemicals is hindered by background contamination and matrix interference. Aggressive extraction protocols may further bias results by artificially enhancing additive release or degrading labile compounds, thereby distorting environmentally relevant emission patterns.^15^ To date, comprehensive screening of complex additive mixtures at environmentally relevant concentrations remains challenging.

Moreover, accurately predicting the environmental fate of these emissions remains a significant challenge. Transport of plastic-derived chemicals to groundwater and surface waters is governed by processes such as advection, dispersion, sorption–desorption, and degradation. Characterizing the mobility of these chemicals requires accounting for the complex interplay of their molecular properties, the geochemical components of the soil profile (e.g., minerals and organic matter), and the spatially and temporally variable flow regimes. Current experimental designs rarely capture these dynamics, as they typically rely on oversimplified systems and a narrow subset of compounds (often at environmentally unrealistic concentrations) that fail to reflect field conditions and leave most plastic-derived chemicals uncharacterized. This undermines the applicability of conventional substance-by-substance approaches and necessitates more integrative, process-based modeling frameworks.

Together, these analytical and mechanistic uncertainties culminate in a significant blind spot in current risk assessment frameworks. By relying on particle-centric paradigms that focus primarily on abundance, size distribution, and physical transport, current management strategies fail to account for the diffuse chemical burden originating from agricultural plastics, which represents a more mobile and often less constrained pathway of exposure to water systems. Current regulatory models are not yet equipped to handle the multidimensional complexity of agricultural plastic pollution, systematically underestimating its long-term impact on aquatic systems. Addressing this mismatch between how environmental exposure from agricultural plastics is conceptualized and how it occurs in practice requires a shift toward holistic assessment frameworks that explicitly link chemical emissions and interfacial processes to water quality impacts.

## Implications for Assessment, Design, and Regulation

The transition from a particle-centric to a chemical-focused perspective necessitates a fundamental shift in how agricultural plastics are assessed, designed, and regulated. First, environmental monitoring and risk assessment must move beyond particle-based metrics toward quantifying chemical emissions and fluxes under realistic field conditions. This includes generating long-term, field-relevant data on additive release and transformation products, rather than relying on short-term laboratory extractions that fail to capture diffusion-controlled processes and the temporally decoupled nature of emissions under variable hydrological conditions. Second, material design must prioritize chemical composition, green-chemistry principles, and release dynamics and transformation behavior as central criteria for sustainability. Agricultural plastics are not inert materials but engineered chemical systems whose performance and environmental behavior are defined by their additive composition. This requires greater transparency in additive formulations and the adoption of safe-by-design principles that reduce hazardous and persistent additives and limit their emission potential and mobility, while avoiding regrettable substitutions. Importantly, the transition to biodegradable materials alone should not be assumed to reduce environmental risk, as faster degradation can alter emission dynamics, shifting from slow, continuous, low-level release toward more rapid and temporally concentrated pulses of mobile plastic-derived chemicals. Third, regulatory frameworks must expand beyond polymer persistence and biodegradability to explicitly address chemical emissions from agricultural plastics, moving toward emission- and flux-based approaches that consider the release, transport, and environmental fate of plastic-derived chemicals, analogous to existing frameworks for organic micropollutants. This includes incorporating additive-related hazards into environmental standards and developing strategies to manage diffuse, non-point chemical inputs, despite the vast, largely uncharacterized chemical space associated with plastic additives. In addition, risk assessment should account for the combined effects of particulate residues and dissolved chemical mixtures across interconnected soil–water systems. This requires moving beyond single-substance assessments to address mixture toxicity, as plastic-derived chemicals are released as dynamic combinations of additives and transformation products whose cumulative effects cannot be inferred from individual compounds alone. Together, these steps would align material innovation, environmental monitoring, and regulation with the dominant pathways of exposure, providing a mechanistic basis for protecting groundwater, surface waters, and drinking water resources from agricultural plastic-derived chemical contamination.
